# Preparatory Effects of Distractor Suppression: Evidence from Visual Cortex

**DOI:** 10.1371/journal.pone.0027700

**Published:** 2011-12-02

**Authors:** Jaap Munneke, Dirk J. Heslenfeld, W. Martin Usrey, Jan Theeuwes, George R. Mangun

**Affiliations:** 1 Department of Cognitive Psychology, VU University Amsterdam, Amsterdam, The Netherlands; 2 Department of Neurobiology, Physiology and Behavior, University of California Davis, Davis, California, United States of America; 3 Department of Neurology, University of California Davis, Davis, California, United States of America; 4 Center for Neuroscience, University of California Davis, Davis, California, United States of America; 5 Center for Mind and Brain, University of California Davis, Davis, California, United States of America; 6 Department of Psychology, University of California Davis, Davis, California, United States of America; University of British Columbia, Canada

## Abstract

Spatial selective attention is the mechanism that facilitates the selection of relevant information over irrelevant information in the visual field. The current study investigated whether foreknowledge of the presence or absence of distractors surrounding an impending target stimulus results in preparatory changes in visual cortex. We cued the location of the target and the presence or absence of distractors surrounding the target while changes in blood oxygen level dependent (BOLD) signals were measured. In line with prior work, we found that top-down spatial attention resulted in an increased contralateral BOLD response, evoked by the cue throughout early visual cortex (areas V1, V2 and V3). In addition, cues indicating distractor presence evoked a substantial increase in the magnitude of the BOLD signal in visual area V3, but not in V2 or V1. This study shows that prior knowledge concerning the presence of a distractor results in enhanced attentional modulation of visual cortex, in visual areas where neuronal receptive fields are large enough to encompass both targets and distractors. We interpret these findings as evidence that top-down attentional control processes include active preparatory suppression mechanisms for irrelevant, distracting information in the visual scene.

## Introduction

A visual scene contains large amounts of information, only a subset of which may be relevant for our current behavioral goals. The abundance of distracting information in a given scene calls for a mechanism that effectively separates relevant from irrelevant information. Visual selective attention is the ability of organisms to differentially process and act upon relevant versus irrelevant information in vision [1].

Previous research has shown that selective attention can operate in a spatially specific manner, acting at specific locations in the visual field that contain relevant stimuli [2]–[4]. Numerous reports have supported the view that information processing at attended locations is facilitated, demonstrating improved detection and discrimination of attended-location events (e.g. [5], [6]). The neural correlates of visual spatial selective attention have been studied extensively. Results of human functional imaging studies have shown increased patterns of activity in striate and extrastriate cortex as a result of top-down allocation of attention [7]–[12]. The increased activity in visual cortex is assumed to reflect enhanced processing of visual information as a result of the selective allocation of attention.

In addition to the enhanced processing of information presented at relevant locations, a number of studies have focused on possible suppressive effects of attention on irrelevant information and locations. The suppressive influence of selective attention can be observed in studies showing that processing of information presented close to the focus of attention gets attenuated compared to items presented further away from this location [13]–[17]. However, in these studies, the suppressive mechanism of attention is always dependent on where attention is focused and seems to serve as a by-product of attentional focusing, increasing the signal-to-noise ratio between the target location and the surrounding area. This raises the question of whether or not participants have top-down control over this suppressive mechanism in terms of where and when it is directed.

Contrary to an automatic suppression of items presented around the focus of attention, the suppressive nature of attention may be able to be controlled in a top-down manner in response to conditions where distractors are anticipated. Studies have shown increased spatial cueing effects (i.e., the difference in accuracy or reaction times when possible target locations are validly or invalidly cued) in conditions in which a target was surrounded by irrelevant information compared to trials in which only a target was presented [18]–[20]. Thus, it appears that selection of relevant information may to a certain extent be dependent on the ability to suppress irrelevant information in a visual setting.

Spatially specific attentional modulation of visual cortex can be observed prior to the presentation of a target stimulus when a cue directs attention towards the location of the upcoming target [12], [21], [22]. These increases in background neural activity due to preparatory attention have been coined baseline shifts [23]. A question that has not been fully addressed concerns whether a similar mechanism can suppress the location of irrelevant information if an observer is informed where to expect this information. That is, can preparatory attention specify not only which stimuli should receive enhanced processing, but also which are subject to inhibition? Would such processes (facilitation and suppression) both act during preparatory attention, or might bottom-up information in the scene be required to engage modulatory, for example, suppression of distractors?

Recently, it has been shown that attentional modulation may reflect processes related to expected distractor properties, such as their location in the visual field or their presence or absence on a given trial [18], [24]–[26]. Furthermore, some of these effects have been observed prior to the onset of the visual information, as changes in baseline activity [26]. Using functional magnetic resonance imaging (fMRI), Serences and colleagues showed that knowledge concerning the likelihood of distractor presence influenced baseline signals in visual cortex. On each trial, the location of two target digits was cued, and the likelihood that targets would be accompanied by distractors was manipulated. The behavioral data of Serences et al. showed that participants scored significantly better on distractor-present trials when distractors were expected compared to when distractors were unexpected. Importantly, when distractors were absent, distractor cueing had no effect. Their fMRI data showed an increased neural response in early visual cortex (including V1) for attended locations compared to unattended locations, and this was largely caused by cue-evoked responses in trials when distractors were expected. The fMRI data were thus in line with the behavioral data, showing that preparatory effects of attention were larger in conditions in which the level of distractor suppression was higher. A related study using event-related potentials (ERPs) also provided evidence for preparatory changes in brain activity when subjects expected an upcoming distractor [27]. In addition, Ruff and Driver [25] showed an increase in BOLD signal in visual cortex at sites that coded the location of an expected upcoming distractor stimulus. These results indicate that distractor expectancy can lead to an increase in BOLD signal, rather than a decrease as observed in center surround models of attention [14], [28].

**Figure 1:**
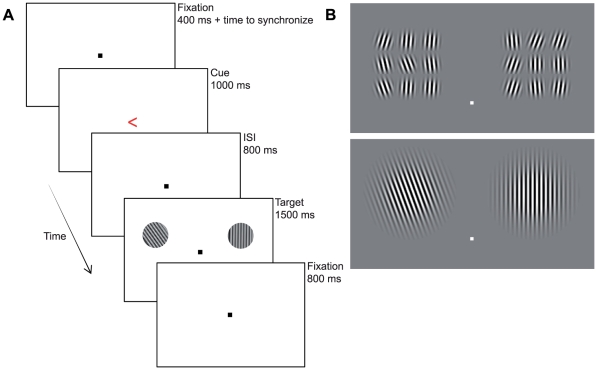
Time course and stimuli of a typical experimental trial. A) Participants focused on a central fixation point until a cue appeared. The cue indicated the visual hemifield in which the target would appear (arrow direction), as well as whether or not the target would be surrounded by distractors (arrow color). Participants responded to the orientation of the Gabor patch in the cued location only. B) Examples of actual target and distractor displays. The top-panel shows target and distractors, the target always being the middle patch on the cued side. The bottom panel shows the target display when no distractors were present.

The current study investigates the neural mechanisms of preparatory distractor suppression in early visual cortex. In line with Ruff and Driver [25] we expect an increase in BOLD signal at neural sites that code the location of the expected distractor. Similar to Serences et al. [26], we used a paradigm in which a target was surrounded by distractors in close proximity. However, the current study differs from the study of Serences and colleagues in two important ways. First, Serences et al. investigated how neural activity at the attended target location changed as a result of distractor likelihood. The study did not investigate neural activity at the distractor locations directly. Therefore, the study by Serences et al. only discusses how target-related neural processes are changed by the likelihood of upcoming distractors surrounding the target. In the current study we used a design in which the visual area stimulated during distractor trials was similar to target-only trials (see Figure 1). That is, we used a design in which the cued location always had approximately the same size regardless whether distractors were present or not. This allowed us to study the cue-evoked neural modulation at the location where targets and distractors were expected. Second, Serences et al. always cued two target locations in the visual field, likely requiring divided attention in order to observe both targets. We cued a single target location in order to investigate the classic effects of visual spatial selective attention.

## Materials and Methods

### Participants

Twelve paid volunteers participated in the experiment (mean age: 27.2 years, 8 males). All participants were healthy, had normal or corrected-to-normal vision, and were right handed. Data from one participant was removed due to technical difficulties. All analyses are based on the remaining eleven participants. The experimental procedure was conducted following the guidelines laid down in the Helsinki Declaration and was approved by the human subject review board of the University of California, Davis. All participants gave written informed consent prior to the start of the experiment.

### Stimuli and Task

Participants performed a spatial cueing task in which the to-be-attended location and the likelihood of distractors were both cued on a trial-by-trial basis. Figure 1 shows a typical experimental trial. At the start of a trial, participants fixated a centrally presented fixation point. An attentional cue, a left or right pointing colored arrow (subtending a visual angle of 0.6×1.0 degrees) was then presented at fixation. The arrow direction (left vs. right) instructed the subjects where to focus covert attention (without eye movements) for that trial. The arrow color (blue vs. red - counterbalanced over participants) informed the participants whether or not the stimulus would be surrounded by distractors. The colored arrow cue was presented for 1000 ms, followed by a blank screen for 800 ms. Subsequently, a bilateral stimulus array was presented, remaining on the screen for 1500 ms. After target offset, only the fixation point remained onscreen until the next cue was presented 2100 ms later. Participants were instructed to make a differential response to the orientation of the target Gabor patch, which could be either tilted to the left or the right from a perfectly vertical orientation.

#### Distractor Absent Arrays

When no distractors were present (Figure 1B, bottom), the target consisting of a tilted black and white Gabor patch (subtending a visual angle of 3.5 degrees in diameter) was presented above the horizontal meridian (1.75 degrees to the center of the patch). The Gabor was placed with its center at a distance of 3 degrees of visual angle from the fixation marker. A non-target (not tilted) patch of the same size was presented at the corresponding location in the opposite hemifield.

#### Distractor Present Arrays

When distractors were present (Figure 1B, top), the target was a small Gabor patch, surrounded by eight patches of equal size (0.8 degrees per patch) and was placed at the same distance from fixation compared to the large target patch. At the same time nine small non-target/distractor patches were placed at the corresponding location in the opposite hemifield. All Gabor patches had a spatial frequency of 6.3 cycles per degree. The patches could be tilted either to the left or the right, the angle of orientation depending on the performance of the participant (see *Staircase Procedure*). Participants responded to the orientation of the target patch by pressing a button with the right or left hand.

In order to isolate cue-related BOLD signals, 41% of all trials consisted only of the presentation of a cue, and were not followed by a target display (cue-only trials). Furthermore, 18% of the trials were blank in which neither cue nor target was presented (null trials). The remaining 41% of the trials consisted of cue + target trials. The order of different trial-types was semi-random and was designed in order to optimize independency between the event-related signals. Altogether, these aspects of the design permitted the cue and stimulus array evoked BOLD signals to be deconvolved [29], [30]. In total, 384 trials were presented to each participant, divided over 6 blocks. Stimulus presentation and response collection were controlled using E-Prime 2.0 (Psychology Software Tools).

### Staircase procedure

Because presenting targets with or without distractors may lead to differences in task difficulty between these two trial-types, a staircase procedure was developed to equate differences in task difficulty. Changes in task-difficulty were accomplished by adjusting the angle of orientation of the target stimuli based on the performance of the participant. A moving average was calculated of the participants' average performance over the last 4 trials separately for distractor-present and distractor-absent trials. If the average performance over the last 4 trials dropped below 75% correct, the angle of orientation from vertical (defined as 90 degrees) was increased by 1 degree, thereby increasing the discriminability of the target. When the participants' performance rose above 75% the angle of orientation was decreased by 1 degree (getting closer to a vertical orientation), making the task more difficult. When the angle of orientation deviated by only 1 degree from a vertical orientation (i.e. 89 or 91 degrees), increments of 0.1 degree were used to increase or decrease task difficulty. Separate performance levels were calculated for target-only and target + distractor trials, to obtain approximately 75% correct responses in either condition.

### Scan Acquisition

Images were collected on a 3T Siemens TRIO scanner (Siemens Medical Systems, Erlangen, Germany) at the UC Davis Imaging Research Center. Participants viewed the stimuli through a mirror, attached at a 45 degree angle to the head coil. The experiment was back-projected on a semi-transparent screen placed outside the bore, using a 75 Hz Digital Projection Mercury 5000 HD projector. All subsequent analyses of fMRI data were performed using BrainVoyager 2.1 (Brain Innovation, Maastricht, The Netherlands).

Scanning acquisition parameters for the main experimental task were: TR = 1800 ms, TE = 25 ms, flip angle = 80°, slice thickness = 3.6 mm, slice gap = 0 mm (no gap), acquisition matrix = 64×64, in-plane resolution = 3.2×3.2 mm. Functional data were collected using a gradient recalled EPI sequence scanning the whole brain in 33 near-axial slices. A 3-D anatomical scan was made at the end of the session, using a T1-weighted MP-Rage sequence. Scanning parameters were: TR = 1660 ms, TE = 2.17, TI = 1100 ms, flip angle = 8°, sagittal slice thickness = 1 mm, acquisition matrix = 256×256 pixels, in-plane resolution = 1×1 mm.

### Retinotopic mapping of visual areas

Mapping the borders of early visual areas (V1–V3) was accomplished by presenting a slowly rotating bifield checkerboard wedge pattern (see [31]). The wedges, with a width of 30 degrees, completed eight full rotations (meaning that both hemifields were fully stimulated twice on each rotation; i.e. 16 times), each rotation lasting 48.5 seconds (24 TRs, each TR, 2020 ms). The checkerboard pattern flickered at 9 Hz.

**Figure 2:**
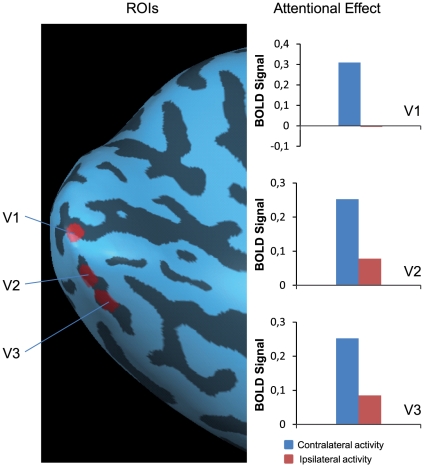
Example of mapped regions-of-interest (ROIs) and the attentional effect obtained at these ROIs. Example of ROIs mapped onto the inflated left hemisphere of a typical subject. Bar graphs represent the attentional effect (contra-lateral (blue) activity versus ipsilateral (red) activity) obtained at these ROIs. For each participant and each hemisphere ROIs were mapped in this manner and time courses were obtained at these individually mapped ROIs.

An additional localizer task was employed to identify target locations within regions of early visual cortex. Circular checkerboard patterns (9 Hz) with a diameter of 3.5 degrees were presented at the left and right stimulus locations used in the attention task. The checkerboard patterns had the same size as the distractor-absent Gabor patches in the main experiment (Figure 1B, bottom). Each pattern was presented with a duration of 2020 ms (1 TR) after which a blank screen with a duration of either 4040 ms (2 TR) or 6060 (3 TR) was presented before the next pattern would appear. Checkerboard patterns were presented at the target locations in a semi-random order. This localizer procedure, combined with the bifield wedge stimulation, pinpointed the projections of target and distractor locations in early visual areas. For illustrative purposes ROIs in the left hemisphere of a single-subject, as defined by activity obtained during the localizer tasks, are presented in Figure 2. Furthermore, the bar graphs in Figure 2 show the attentional effect (for illustrative purposes) obtained at ROIs defined for this specific participant. That is, contralateral activity was derived after an attention directing cue to the right visual hemifield, whereas ipsilateral activity was observed after a leftward pointing cue, averaged over distractor conditions.

### MRI Data Analysis

The first two volumes of each block were omitted in order to avoid differences in T1 saturation. The preprocessing of the remaining functional volumes consisted of motion correction, slice scan-time correction, high-pass filtering (0.01 Hz), spatial smoothing (3 mm FWHM Gaussian kernel); no temporal smoothing was employed.

After preprocessing, all functional scans of each participant were automatically and where necessary manually co-registered to the anatomical scan of that participant, aligning the functional with the anatomical data in 3 dimensions. Both functional and anatomical data were subsequently converted to Talairach space [32]. Converting functional data to Talairach space resulted in 4D functional data sets (e.g. [33]). Anatomical data was automatically, and where necessary, manually segmented in order to separate the different tissues of the brain. Based on the observed gray and white matter boundary, a model of the cortical mantle of each hemisphere was created. This model was subsequently inflated resulting in a smooth reconstruction of the cortical surface on which cortical gyri and sulci were displayed. Regions of interest (ROIs) derived from the visual mapping experiments were defined for each of the 24 hemispheres in ventral visual areas V1v, V2v and V3v/VP (henceforth called V1, V2 and V3).

In order to investigate the effects of distractor cueing on neural activity in visual cortex, two types of analysis were used. First, an event-related averaging procedure was used in which the cue-only response was subtracted from the cue + target response. This analysis could provide a measure of how target processing was influenced by the different cue types (distractor present or distractor absent). However, this analysis did not have enough power to reliably analyze attentional modulation of the target stimuli. Therefore, the results in this paper only address cue-induced BOLD signals.

**Figure 3:**
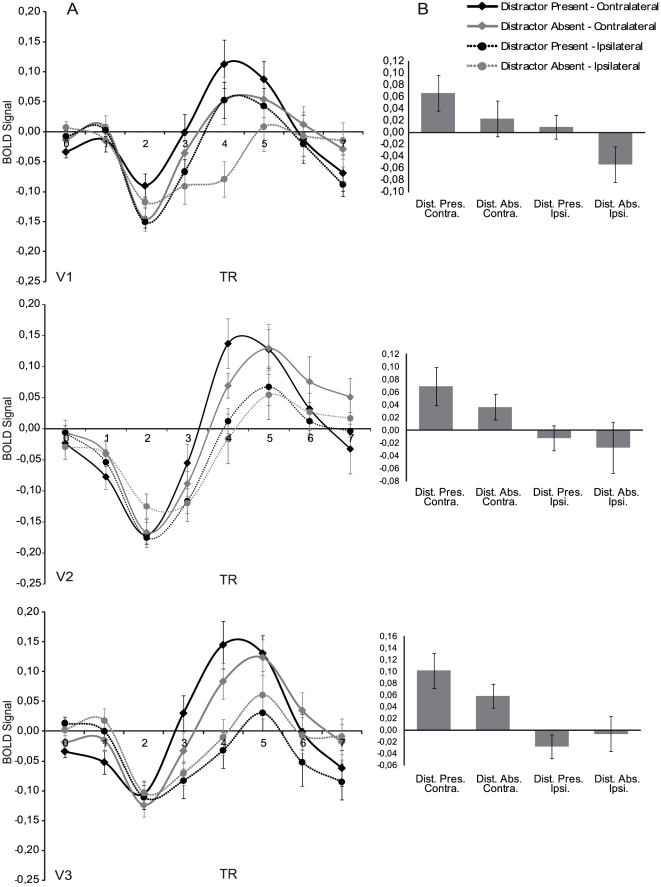
Obtained time courses and averaged BOLD signal. A) Deconvolved time courses of the four trial types measured from cue onset. B) Average signal calculated over the peak of the BOLD response (TR3 – TR5; 1 TR = 1.8 s). Regions of visual cortex contralateral to the cued visual hemifield showed larger responses compared to ipsilateral regions in all ROIs during this interval. There was no main effect of distractor expectation, but in V3, an interaction between laterality and distractor expectation was observed for this interval, showing that preparation for a distractor had the largest effect at contralateral sites. Error-bars reflect standard-error corrected for the use of a within-subjects design.

Second, BOLD responses were estimated to all cues, independent of whether they were followed by a target or not. A deconvolution General Linear Model (GLM) was employed using predictors for cues and (if present) targets, thereby separating cue-related activity from BOLD changes due to subsequent target presentation. A regressor was assigned to each of 8 volumes following the onset of target displays, as well as all leftward and rightward pointing cues, separately for cues that indicated the presence of distractors and cues that indicated that no distractors would be presented. These analyses were performed separately for each participant and ROI, and the resulting time-series of response estimates for the cues were averaged over hemispheres for each ROI and condition. These cue-evoked response estimates were further investigated in two ways. First, we investigated whether attention modulated visual cortex in a spatially specific way by comparing cue-evoked activity in the hemisphere contralateral to the cued location to the evoked activity in the hemisphere ipsilateral to the cued location. The term hemisphere in this regard, refers to the appropriate ROI within the hemisphere. Second, in order to investigate preparatory effects of distractor expectation, the response estimates evoked by cues indicating the presence or absence of distractors were compared. Differences between these conditions were expected to be maximal when the magnitude of the BOLD signal was largest. Therefore all further analyses will focus on the time period reflecting this part of the BOLD response, which in the current experiment is between 5.4 and 10.8 seconds after cue onset (see Figure 3).

## Results

### Behavioral results

Accuracy was measured separately for distractor present and distractor absent trials. The task was designed in such a way that no differences in difficulty should be able to explain observed neural responses. Indeed, behavioral data indicated that performance on both trial types was equivalent (distractor present trials 75.3% correct, distractor absent trials 74.9% correct). A paired samples t-test found no significant differences between these trial types (*t*(10) = 0.405, *p* = 0.694). Therefore, any observed difference in neural response cannot be attributed to differences in task difficulty, but may instead be attributed to the experimental manipulations of interest in the design.

As described in the Methods, task difficulty was continuously adjusted for each subject by increasing or decreasing the angle of the target patch relative to vertical, as a function of a running average of the subject's performance. On average, this procedure led to the angles of the targets deviating from vertical by 8 degrees for the distractor present trials, and 1 degree for the distractor absent trials.

### fMRI Data

By means of a deconvolution analysis, changes in the hemodynamic response as a result of attention-directing cues were calculated separately for the attended (contralateral) and unattended (ipsilateral) locations, and for distractor present and distractor absent conditions. These analyses were performed separately for each participant and ROI. Figure 3A shows the evoked time-courses for V1, V2 and V3 for the four trial types, averaged over participants and hemispheres. The differences between these time courses were statistically tested for TR 3 to TR 5 (5.4–10.8 s after cue onset) reflecting the maximal amplitude of the BOLD signal (see Figure 3B).

An ANOVA with preparatory attention direction (ROIs contralateral vs. ipsilateral to the attended location) and distractor expectation (distractor present vs. distractor absent) as within-subject factors was performed investigating the effects of directing spatial attention and the expectation of distractor presence or absence on the obtained fMRI signals. The ANOVA showed a main effect of preparatory attention direction, indicating that the magnitude of the BOLD response was larger at ROIs contralateral versus ipsilateral to the attended hemifield. This effect was obtained for all ROIs (V1: *F*(1,10) = 6.779, *p* = 0.026; V2: *F*(1,10) = 7.636, *p* = 0.020; V3: *F*(1,10) = 15.527, *p* = 0.003), which shows that spatial attention was deployed towards the cued location.

The effect of distractor expectation was investigated during the same time period in which effects of spatial attention were observed. No main effect of distractor expectation was observed in any of the ROIs (V1: *F*(1,10) = 1.164, *p* = 0.306; V2: *F*<1, *ns*; V3: *F*<1, *ns*). However, a significant interaction between distractor expectation and preparatory attention direction was observed in V3, showing a larger difference between contra- and ipsilateral preparatory BOLD signal when the presence of distractors was precued, compared to when the cue indicated that no distractors would be present (*F*(1,10) = 6.564, *p* = 0.028). This effect was not observed in V1 (F<1, *ns*) or V2 (F<1, *ns*). Taken together, these results indicate that cueing distractor presence only influenced the hemodynamic response in V3, and that this effect is attentional in nature as indicated by an increased difference between contra- and ipsilateral BOLD signals.

**Figure 4:**
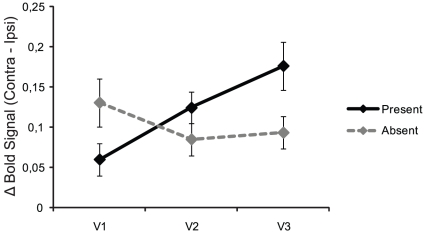
Effects of attention (contralateral – ipsilateral BOLD responses) for distractor present and absent trials. The data points reflect averaged BOLD responses over the two hemispheres for TR = 4, reflecting the peak of the BOLD signal. When distractors were expected, the effect of attention differs in the early visual areas, increasing linearly over higher visual areas (black line). This effect was not observed when distractors were not expected (gray line). Error-bars reflect standard-error corrected for the use of a within-subjects design.

Additionally, planned comparisons regarding the influence of distractor preparation on the BOLD signal in visual cortex showed that the size of the attention effect (the difference between contra- and ipsilateral BOLD signals) changed as one moves up the visual stream from V1 to V3. This change was observed at the peak of the BOLD response (TR = 4) and was found to be larger when distractors were expected compared to when they were not expected as indicated by a significant 3-way interaction (ROI×preparatory attentional direction×distractor expectation: *F*(2,20) = 6.104, *p* = 0.009). Similar to aforementioned analyses, the effect of attention on visual cortical processing was defined as the difference between contra- and ipsilateral activity as this difference is assumed to measure the added effect of attention on visual cortical processing at contralateral sites compared to ipsilateral sites. Figure 4 shows the difference in attentional effect separately for distractor present and distractor absent trials. For distractor present trials, a strong linear trend is observed between the activation pattern from V1 to V3 (linear trend: *F*(1,10) = 7.288, *p* = 0.022), supported by the interaction between ROI and preparatory attentional direction for distractor present trials (*F*(2,20) = 5.009, *p* = 0.017). A paired-samples t-test showed that this interaction was caused by a larger attentional effect in V3 compared to V1 (*t*(10) = 2.700, *p* = 0.022). Neither the linear trend (*F*<1, *ns*), nor the interaction was observed for the distractor absent trials (*F*<1, *ns*). This suggests that only when distractors are expected, does attention differentially modulate the individual regions in early visual cortex.

## Discussion

We found that endogenous cues evoked hemodynamic modulations throughout early visual cortex, including V1, V2 and V3. These findings replicate and extend earlier findings showing that preparatory attention acts upon spatially specific regions of early visual cortex [12], [21], [22], [34]–[36]. More importantly, the present study also shows that a cue indicating that distractors will be present on the upcoming trial resulted in a larger preparatory attentional effect (the difference between contra- and ipsilateral BOLD signal magnitude) than a condition in which the cue indicated that distractors would not be present. This effect was statistically reliable only in regions of V3 contralateral to the cued target location (compared to the ipsilateral side). Since no main effect of distractor presence was observed in any of the ROIs, it can be assumed that neural modulation due to distractor expectation was not the result of more general neural processes such as increased arousal. Instead the effect was observed only in V3 and was retinotopic in the sense that it was larger contralateral compared to ipsilateral to the attended location. This indicates that neural preparatory attention processes in V3 are not only modulated by prior knowledge concerning the location of the upcoming target, but also by the characteristics of that target. More specifically we show that knowing that the target will be accompanied by distractors results in a change in the BOLD response, enhancing the spatial cueing effect.

Why was modulation of the preparatory BOLD response, which was evoked by prior knowledge regarding distractor presence, only found in V3 and not in V1 and V2? One possible explanation is that the sizes of receptive fields in early visual areas are so small that the target and distractor used in the current study would fall in separate receptive fields in these early visual areas but not in later areas such as V3 [37]. Receptive field sizes are known to increase in higher visual regions [38]. Previous work has shown that when multiple stimuli are present in the receptive field of a neuron, they compete for neural representation (i.e., biased competition). It is hypothesized that attention resolves the competition between multiple items presented within a neuron's receptive field [21], [39]–[41]. Competition between multiple items is resolved by focusing attention on the relevant target stimulus, thereby attenuating the interfering effects the irrelevant stimulus has on target processing. Therefore, when multiple items are presented in the visual field, irrelevant items may be suppressed, but only when these items are presented in the same receptive field.

Because the effects observed in the current study represent preparatory processes induced by the cue, it indicates that processes related to distractor processing were engaged in a top-down manner. This indicates that the cue may induce a top-down process that prepares for the competition in the upcoming trial. We show that only when distractors are expected, is there significant differential modulation of extrastriate regions in visual cortex (area V3). This effect is not observed when distractors are not expected. Importantly, an effect of attention is still observed in the absence of distractors caused by the signal enhancing quality of visual attention [42]. Note that these effects cannot be explained by eye movements. Eye movements would have moved the relevant locations out of the cortical regions of interest, so this would only have weakened (rather than inflated) these effects. The highly significant difference between activation in regions contralateral vs. ipsilateral to the cued side indicates that this was not the case. In addition, the staircase procedure ensured that the number of correct responses was equal in all conditions. This implies that conditions were comparable in terms of visual task difficulty and, hence, the need for eye movements.

The present results are in line with a biased competition model of attention. Receptive field sizes in early visual cortex at an eccentricity of 3° have been approximated at 0.5°, 1.5° and 2.5°–3° respectively in V1, V2 and V3 [38], [43]. The current distractor array subtends a visual angle of approximately 3.2°, the larger part falling in the RF of individual neurons in V3, but not (or at least to a much lesser extent) in the RFs of individual neurons in V2 and V1. Therefore, only in V3 do target and distractors vie for neural representation, resulting in an enhanced attentional response evoked by the need for suppression of distractors. In V1 and V2, target and distractors are coded by different neurons, and prior research has shown that suppression is not required to effectively process the attended target in this situation [40].

Furthermore, the current results show that the attentional effect increases when moving up the visual stream, but only when the target is expected to be surrounded by distractors. Prior studies have shown similar effects. For example, Kastner [44] observed an increase in the magnitude of the attentional effect when proceeding higher up the visual pathway, in a task in which a target stimulus could be surrounded by distractors or presented alone. One crucial difference between the study by Kastner et al. and the results of the current study is that Kastner and colleagues observed this increase in both target conditions with or without distractors. However, the current data reflect cue-induced attentional responses, whereas the study by Kastner et al. shows a direct influence of spatial attention on visual processing of the target and distractors. It is yet unclear whether these two effects reflect a single mechanism or qualitatively different attentional processes. Nonetheless, the current study and the study by Kastner and colleagues show that distractor suppression is not merely a side effect observed at regions in visual cortex that code unattended locations while attention is deployed elsewhere [11], [36], [45].

Further evidence for top-down control of distractor suppression comes from a study by Ruff and Driver [25]. Ruff and Driver employed a paradigm in which both the location of a target was cued as well as the presence or absence of a distractor. In order to separate target and distractor evoked neural responses, target and distractor were presented in opposite hemifields. Ruff and Driver showed that cueing the expectancy of a distractor resulted in a behavioral advantage in target selection in terms of faster response times. Moreover, this effect was only significant when a distractor followed the cue, but not when the distractor was absent. FMRI data of the study by Ruff and Driver showed a preparatory increase in BOLD response between expected distractor presence compared to expected distractor absence at regions of the visual cortex contralateral to the indicated distractor location. These preparatory increases were observed in striate and extrastriate regions of the visual cortex (Brodmann's area (BA) 17, 18 and 19). Under these conditions, no additional modulation reflecting target preparation was observed in regions of the visual cortex contralateral to the cued target location. The results of Ruff and Driver are in line with the current study in so far as both studies show top-down control over a distractor suppressing mechanism and in the finding that the expectancy of distractor presence results in an increase in BOLD signal at regions coding the distractor locations. The observed preparatory increases in BOLD signal in striate and extrastriate visual cortex (BA 17, 18 and 19) cannot be explained by competition between the target and the distractor, because receptive field sizes of neurons at these levels of visual cortex do not encompass the entire visual field.

A possible explanation for the effects observed by Ruff and Driver [25] is that the increased BOLD response may reflect occipital “predictive coding” of the pattern of expected stimulation in visual cortex (see [46]), assuming that neurons in the visual cortex are activated already by an expected pattern of stimulation. Note that this holds for both target and distractor stimuli. However, an explanation in terms of “predictive coding” is unlikely to apply to the current data, as this effect of expectation should have propagated down from V3 to V2 and V1. Nevertheless, an increase in BOLD signal, as obtained by Ruff and Driver, as well as in the current study, suggest a mechanism of distractor suppression that is clearly different from suppression effects as observed in center-surround models, where suppression is accompanied by a decrease in neural activity surrounding the locus of attention [14], [28].

Although the current study shows cue induced patterns of activation for spatially selective attention and distractor suppression, no inferences are made as to how these preparatory effects influence target processing. Due to the fast-event related nature of the study and the absence of target-only trials, modulation of the target and surrounding distractors as a result of different preparatory attentional processes could not be measured independently. Therefore, the current study provides only tentative evidence that the subsequent processing of targets and distractors are modulated by attention per se. Indeed, contrary to studies showing enhanced visual processing as a direct result of preparatory attention (e.g. [21]), other studies have shown that preparatory attention is not always followed by increased neural processes underlying attentional modulation of visual events (e.g. [23]). For this reason, the conclusions in the current study cannot be generalized beyond the findings that cue induced effects of spatially selective attention can be observed in V1–V3, whereas cue induced attentional suppression acts only on area V3. A more elaborate experimental design is required to investigate how these attentional effects modulate the neural processes underlying target selection and processing.

In conclusion, the current study shows separable effects of preparatory spatial selective attention and distractor suppression in visual cortex. These effects were observed to interact as information moves up the visual hierarchy from V1 to V3, in line with the changing receptive field sizes of neurons in these areas and the spatial extent of distractors and targets in this study. These findings may be interpreted within a modified biased competition account of attention, in which interfering influences of irrelevant information are suppressed by a preparatory top-down signal from the attentional control system that enables efficient distractor suppression within visual cortex.
